# Undernutrition impairs the quality of growth plate and trabecular and cortical bones in growing rats^1^

**DOI:** 10.1590/s0102-865020190030000001

**Published:** 2019-03-18

**Authors:** Patrícia Madalena San Gregório Guedes, Ariane Zamarioli, Iara Inácio Botega, Raquel Assed Bezerra da Silva, João Paulo Mardegan Issa, Mariana Maloste Butezloff, Yara Terezinha Corrêa Silva Sousa, João Paulo Bianchi Ximenez, José Batista Volpon

**Affiliations:** I Fellow Master degree, Postgraduate Program in Health Sciences Applied to the Locomotor System, School of Medicine, Universidade de São Paulo (USP), Ribeirao Preto-SP, Brazil. Design of the study, technical procedures, acquisition and analysis of data, manuscript preparation.; II Researcher, Laboratory of Bioengineering, School of Medicine, USP, Ribeirao Preto-SP, Brazil. Interpretation and analysis of data, critical revision.; III Fellow Master degree, Postgraduate Program in Health Sciences Applied to the Locomotor System, School of Medicine, USP, Ribeirao Preto-SP, Brazil. Technical procedures, acquisition of data.; IV PhD, Associate Professor, Department of Children’s Clinic, School of Dentistry, USP, Ribeirao Preto-SP, Brazil. Acquisition of data, critical revision.; V PhD, Associate Professor, Department of Morphology, Physiology and Basic Pathology, School of Dentistry, USP, Ribeirao Preto-SP, Brazil. Analysis of data, critical revision.; VI Fellow PhD degree, Postgraduate Program in Health Sciences Applied to the Locomotor System, School of Medicine, USP, Ribeirao Preto-SP, Brazil. Technical procedures, acquisition of data.; VII PhD, School of Dentistry, Universidade de Ribeirão Preto (UNAERP), Brazil. Interpretation of data, critical revision.; VIII Fellow PhD degree, Postgraduate Program in Toxicology, School of Pharmaceutical Sciences, USP, Ribeirao Preto-SP, Brazil. Statistical analysis.; IX Full Professor, Department of Biomechanics, Medicine and Rehabilitation of the Locomotor System, School of Medicine, USP, Ribeirao Preto-SP, Brazil. Design, intellectual and scientific content of the study; critical revision; final approval the manuscript.

**Keywords:** Malnutrition, Growth Plate, Cancellous Bone, Cortical Bone, Rats

## Abstract

**Purpose:**

To investigate the effects of dietary restriction on the growth plate and long bone tissue in growing rats.

**Methods:**

Sixty male Wistar rats were randomly assigned to two groups: Control (Con) and Diet-restricted (Res). After weaning, the Res rats were offered 50% of the chow ingested by the control (*ad libitum* food intake). The animals were subdivided into two subgroups with follow-ups up to 56 or 70 days. After euthanasia, the growth plate of tibias was analyzed by histomorphometry, micro-computed tomography, and mechanical test. The trabecular and compact bones were evaluated by histomorphometry, dual-energy X-ray absorptiometry, and micro-computed tomography (μCT). Real-time PCR was used to analyze gene expression.

**Results:**

Although dietary restriction did not alter gene expression, several phenotypic changes were seen in the growth plate; i.e., decrease in volume, reduction in total area and height, decrease in the area ossified zones, mechanical weakening, reduction in mass of trabecular and cortical bone, lower bone density, deterioration of the trabecular and cortical microarchitecture, and trabeculae with lower collagen deposition.

**Conclusion:**

Dietary restriction had severe detrimental effects on the growth plate and trabecular and cortical bone.

## Introduction

Growth plate is a specialized structure which is responsible for the longitudinal growth of long bones as it promotes ossification, thus resulting a cumulative bone deposition at the metaphysis. Growth plate cells proliferate, differentiate, and undergo hypertrophy and apoptosis with replacement by newly formed trabecular bone^1^. During skeletal growth, several structural changes occur to meet the demands during the bone growth period^2^. Therefore, a balanced food intake with specific nutrients such as proteins, minerals, and vitamins are essential not only for the optimum development of skeletal tissues, but also for the maturation of several tissues and organs (i.e., neurological function, and hormonal and endocrine activity)^3^ ^-^ ^7^.

It has been previously shown that an ideal nutritional status during skeletal maturation may be a key factor in achieving an optimum peak bone mass, thus decreasing the incidence of osteoporosis later in life^8^ ^-^ ^10^. Therefore, malnutrition affects the development of long bones and is the major cause of short stature, low weight^11^ and lower peak bone mass^12^ ^,^ ^13^. 

Malnutrition refers to the deficiency, excess, or imbalance in food intake. Undernutrition means the general reduction in the intake of nutrients, while malnutrition refers to the lack of certain nutrients. Previous experimental studies have shown that protein deprivation decreases growth plate thickness and area, and the number of chondrocytes in the hypertrophic and proliferative zones^8^ ^,^ ^14^. The deficiency of vitamin D led to poorer bone density, reduced osteoblastic activity, lower whole bone mechanical integrity, and fewer circulating markers for bone formation (i.e., osteocalcin and IGF-1)^15^ ^,^ ^16^.

Although the depletion of certain nutrients has detrimental impact on the skeletal tissue, the clinical undernutrition often seen in poor people around the world occurs owing to a general reduction in total food intake^14^. Therefore, we reproduced this condition and aimed to investigate the effects of general dietary restriction on growth plate and bone tissue of growing rats. We hypothesized that undernutrition alters the growth plate anatomy and function, thus impairing endochondral bone formation, which results in lower and poor bone quality.

## Methods

###  Animal care 

 The animal experimental protocol complied with the Guide for the Care and Use of Laboratory Animals, and was approved by the Institutional Animal Care and Use Committee of our Institution (protocol 013/2016). 

At 21 days of age, male Wistar rats were weaned and individually housed in metabolic cages under a controlled environment. The metabolic cage does not allow urine and feces accumulation on the floor, thus, permitting to check for the everyday ingested chow. The rats were housed in 55±10% humidity-controlled rooms, and at 23±1°C under a 12-hour artificial light/dark cycle. After allowing three days for adaptation, 60 rats were divided into two groups: Con, weight-matched control rats with unlimited access to food; and Res, rats fed the same diet at 50% of the *ad libitum* food intake by the control group. All rats had free access to water and were assigned to two subgroups according to the experimental follow-ups at 56 days (n=18/subgroup) and 70 days (n=12/subgroup). 

###  Experimental model for undernutrition 

 The control rats were offered chow and water ad libitum, and their daily food ingestion was monitored. Fifty percent of the collective everyday food ingestion of the control animals was offered to the restricted rats and entirely consumed. The inspection of the cage floor guaranteed that all the food offered to these animals was ingested which happened in all cases. A similar protocol has been previously reported in the scientific literature, and was shown to be effective in inducing similar repercussions as those seen in malnourished humans^17^. The animals were weighed three times a week.

###  Euthanasia and sample collection 

 At days 56 or 70, after periods of follow-up (11 weeks old and 13 weeks old, respectively), rats were euthanized with an overdose of sodium thiopental (Tiopental^®^ Cristália, Brazil). The tibias were collected, and cleaned from surrounding soft tissue to allow for morphometric measurements, bone densitometry, tridimensional microarchitecture assessment, histomorphometry, mechanical test, and gene expression. Each tibia was weighed (g) and the length (mm) of the longest mid-diaphysis diameter (mm), and proximal epiphyseal perimeter (mm) was measured (n=7/group). The specimens reserved for mechanical analysis, densitometry, and μCT analysis were kept in 70% cold ethanol. The specimens for histology were fixed in cold 4% paraformaldehyde.

###  Bone densitometry 

 Bone mineral density (BMD) and bone mineral content (BMC) were assessed by Dual-energy X-ray Absorptiometry (DXA) using a Lunar DPX-IQ densitometer (*Lunar; software version* 4.7e, GE Healthcare, UK). After scanning the entire tibia, the BMD and BMC were calculated using a standard region of interest (ROI) 0.09 cm², at the proximal metaphysis, closer to the growth plate. The assessment of the scanning reproducibility (4%) was conducted using the root mean square coefficient of variation.

###  Tridimensional bone microarchitecture 

 Assessment of bone morphology and microarchitecture (μCT) was performed using a SkyScan 1176 (Bruker-microCT, Kontich, Belgium), with a 1-mm-thick aluminum filter. A rotation step of 0,40° with one-frame averaging was selected to obtain an isotropic resolution of 8.5 μm, and the images were reconstructed (NRecon v.1.6.9). The growth plate and trabecular and cortical bone tissues were manually isolated and analyzed using the CTAn software (CTAn v. 1.13.2.1). The total volume of the growth cartilage was measured (TV, mm³). The trabecular microarchitecture in the secondary spongiosa was assessed close to the proximal growth plate, covering a total length of 3 mm. Thus, the bone volume (BV, mm^3^), bone volume fraction (BV/TV, %), trabecular number (Tb.N, 1/mm), trabecular thickness (Tb.Th, mm), connectivity density (Conn.D, 1/mm³), specific bone surface (BS/BV, mm³), and trabecular separation (Tb.Sp, mm) were determined. 

The tibia diaphysis was assessed starting 8.0 mm distally to the proximal growth plate and covering a total length of 2.0 mm. The microstructural parameters determined were cortical volume (Ct.V, mm^3^) and cortical thickness (Ct.Th, mm). Data obtained by μCT are expressed in accordance with standardized nomenclature^18^.

###  Histology 

 The histological analysis was carried out on five left tibias from each group. The bones were fixed in cold 4% paraformaldehyde, decalcified in cold 10% EDTA, and embedded in paraffin, and 5-μm coronal semi-serial sections were obtained. Thus, 72 sections were obtained so that the whole thickness of the proximal tibia metaphysis was cut. After collecting twelve sections, the next ten sections were discarded, and this procedure was repeated for the whole specimen. With this strategy it was possible a randomization of the samples.

For the histomorphometric analysis of growth plate, coronal sections were stained with hematoxylin and eosin (HE). Sections were analyzed under bright field microscopy (Axiovert; Carl Zeiss, Germany), and 50 to 200 magnified images were captured with a CCD camera (AxioCam MRc; Carl Zeiss, Germany). The determination of the total growth plate area (mm^2^), the area of the ossified zone (mm^2^) and the thickness or height (μm) were achieved with Image J software^®^ (NIH, USA). The selection of the areas of interest was made manually. 

For the histomorphometric analysis of the metaphyseal trabeculae, sections that were stained with Masson’s trichrome were examined by bright field microscopy and the images were captured by a digital camera (Zeiss, Germany) with magnifying power of 50x. Using the Axiovision computer software to analyze the images, the blue color was selected in the metaphysis as it represents the newly formed trabeculae and were automatically quantified (B.Ar/T.Ar, %). The Masson’s staining identifies both the newly formed bone and the collagen. The sections were stained with picrosirius red were analyzed under polarized light microscopy and birefringence (AxioImager^®^ Z2, Zeiss, Germany). This permitted the observation of the collagen fibers which appeared in colors orange red (collagen type 1; Col1.Ar/Tt.Ar, %) and yellowish green (collagen type 3; Col3.Ar/Tt.Ar, %). 

###  Growth plate strength testing 

**Figure 1:**
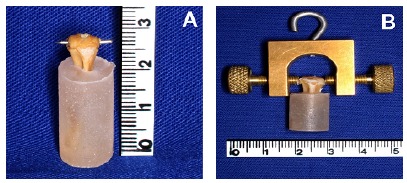
Preparation of the tibia for applying shearing stress to the proximal growth plate. (**A**) Most of the tibia was embedded in a cylindrical acrylic resin that was left exposed only to the proximal segment (metaphysis and epiphysis). A transverse 0.6 mm thick steel wire was transfixed through the ossific nucleus, in the coronal plane. (**B**) A specially designed apparatus was used to connect the wire and the testing machine.

 The growth plate was tested for shearing failure using a universal testing machine with a 50N load cell (EMIC DL10.000, Brazil). For this, the tibia with exception of its proximal end, was embedded in a cylindrical block of acrylic resin (Fig. 1A). Care was taken to avoid overheating during the cement setting by immersing the whole set in cold saline. The proximal epiphysis (ossific nucleus) was transfixed with a 0.6-mm-thick steel-wire in the coronal plane, perpendicular to the longitudinal axis of the bone. A specially designed accessory was made to anchor the wire extremities to the testing machine (Fig. 1B). The acrylic block was fixed in a vise in the horizontal position, and the accessory was connected to the testing machine, so that the vertical traction of the machine was transformed in shearing stress through the growth plate (n=7/group). A constant displacement rate of 10 mm/min was used until growth plate failure. With the software (Tesc 3.04, Brazil), the real-time load-displacement graphs, and the maximal load (N) and stiffness (N/mm) were obtained.

###  RNA isolation and gene expression 

 This analysis was performed only on animals followed up for 56 days. The samples from the proximal metaphysis were immediately stored in a RNAse free recipient, immersed in liquid nitrogen, and kept in freezer at -80^o^C until extraction. For the total RNA extraction from the tibias (n=6/group) was used the Total RNA Isolation System (Promega, Madison, Wisconsin, USA) according to the manufacturer’s instruction. Complementary DNA (cDNA) synthesis was performed with 1 μg RNA, using High Capacity cDNA Reverse Transcription Kit (Applied Biosystems, Foster City, CA, USA) following the manufacturer’s instructions. TaqMan^®^ gene expression assays (Applied Biosystems) were used for quantifying *Col1a1* (assay ID: Rn01463848_m1), *Runx2* (Rn01512300_m1), *Osx* (Rn02769744_s1), and *Sost* (Rn00577971_m1) expression by quantitative PCR on a StepOnePlus PCR machine (Applied Biosystems) and were normalized to the expression of reference gene *GAPDH* (Rn01775763_g1). Samples were run in duplicates, and relative expression was calculated using 2^−ddCT^. The ddCt was calculated as dCt[goi_Res_ - ref_Res_] - dCt[goi_Con_ - ref_Con_], where *goi* is the gene of interest and *ref* is the reference gene. For the descriptive and statistical analyses, ddCT was applied as a continuous variable. Minimum information for publication of quantitative real-time PCR experiments (MIQE) guidelines were followed for interpreting the results of quantitative real-time PCR^19^. 

###  Statistical analysis 

 Continuous variables were expressed as the mean and standard deviations (SD). The results obtained in the groups were compared using t-Test, where p values less than 0.05 were considered to indicate statistically significant differences. All statistical analyses were performed with RStudio 1.0.153 (Rstudio, Inc., USA). GraphPad Prism5^®^ (GraphPad Software, Inc., São Paulo) was used to format the graphs.

## Results

###  Body mass and tibia morphometric parameters 

**Figure 2:**
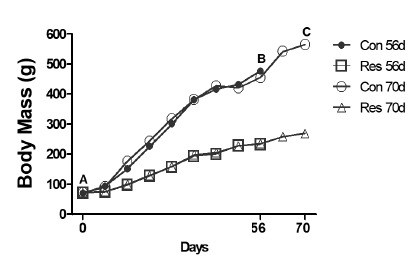
Comparison of body mass (g) among subgroups. (**A**) On day 0 there was no significant difference among the four subgroups (p>0.05). Over time, the diet-restricted animals showed a lower mass acquisition (p<0.05). The 56 (**B**) and 70 days (**C**) represent the days of euthanasia. Con 56: Control group, followed by 56 days; Res 56: Dietary restriction group, followed by 56 days; Con 70: Control group, followed by 70 days; Res 70: Dietary restriction group, followed by 70 days. (Data from the Laboratory of Bioengineering, School of Medicine of Ribeirao Preto, with permission).

 On day zero, there was no difference in body mass among the groups (p>0.05). A time-dependent difference appeared (Fig. 2) showing that the undernourished rats (Res) gained much less body mass than the controls: day 56 (231% versus 580%, p=0.0001), and day 70 (284% versus 707%, p=0.0001). 

Dietary restriction decreased all bone morphometric parameters (length, mass, and perimeter) of tibias (Table 1).

**Table 1:** Morphological parameters of tibias (mean ± SD).

	Con 56	Res 56	Con 70	Res 70
Bone mass (g)	0.88±0.14	0.62±0.076^a^	1.02±0.12	0.63±0.09^b^
Length (mm)	41.63±1.80	38.01±1.31^a^	45.62±1.57	39.31±1.72^b^
Shaft perimeter (mm)	11.68±1.49	9.98±1.11^a^	11.60±0.92	9.78±0.57^b^
Proximal metaphyseal perimeter (mm)	17.99±0.83	15.69±1.87^a^	20.17±2.94	15.03±1.62^b^

^a^p<0.05 vs Con 56; ^b^p<0.05 vs Con 70. Different letters indicate significant statistical difference (p<.05).

Con 56: Control group, followed by 56 days; Res 56: Dietary restriction group, followed by 56 days; Con 70: Control group, followed by 70 days; Res 70: Dietary restriction group, followed by 70 days.

###  Growth plate repercussions of diet ingestion 

 The µCT results showed a not significant decrease in growth plate volume in the undernourished rats, by 39% on day 56 (p=0.06), but a significant decrease by 40% on day 70 in comparison with control rats (p=0.005) (Table 2). 

**Table 2:** µCT analysis of the growth plate, trabecular and cortical bone.

	Con 56		Res 56	Con 70	Res 70
Growth plate Volume (mm³)		6.3±2.1	3.9±0.3 ^a’^	5.5 ±1.2	3.3±0.8 ^b^
Proximal tíbia (trabecular bone)					
BV (mm³)		3.4±1.3	1.4±0.6 ^a^	3.2±1.1	0.8±0.5 ^b^
BV/TV (%)		7.2±2.2	4.2±1.0 ^a^	6.8±2.4	2.8±1.9 ^b^
Tb.Th (mm)		0.05±0.01	0.04±0.01	0.06±0.01	0.05±0.003 ^b^
Tb.N (mm)		1.3±0.2	0.8±0.2 ^a^	1.1±0.4	0.5±0.4 ^b^
Tb.Sp (mm)		0.6±0.1	0.9±0.2 ^a^	1.0±0.4	1.2±0.5
Conn.D (1/mm)		888.9±156.6	208.6±94.0 ^a^	693.3±258.1	235.1±122.1 ^b^
BS/BV (mm)		85.3±9.3	89.1±11.3	76.7±7.9	88.1±7.3 ^b^
Midshaft tibia (cortical bone)					
Ct.V (mm³)		9.3±0.6	6.31±0.7 ^a^	10.0±1.0	5.7±0.9 ^b^
Ct.Th (mm)		0.3±0.01	0.3±0.02	0.3±0.02	0.2±0.01 ^b^

^a^p<0.05 vs Con 56; ^b^p<0.05 vs Con 70. Different letters indicate significant statistical difference (p<0.05).

^a’^p<0.06 vs Con 56. ^a’^ Indicates a tendency to statistical difference.

Con 56: Control group, followed by 56 days; Res 56: Dietary restriction group, followed by 56 days; Con 70: Control group, followed by 70 days; Res 70: Dietary restriction group, followed by 70 days.

**Figure 3:**
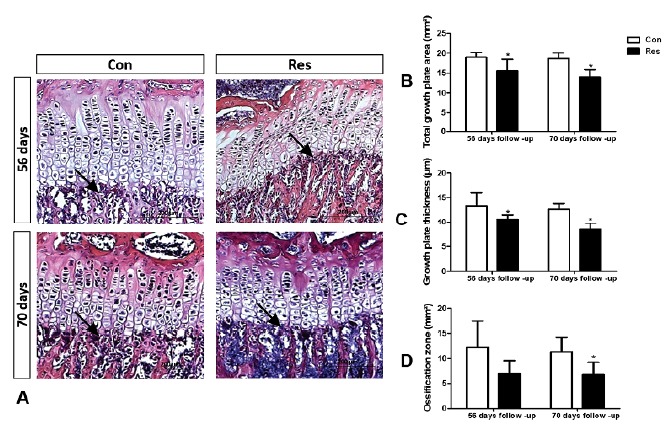
(**A**) Ilustrative histological sections of the proximal growth plates of tibias and comparison of the morphological parameters (B, C, and D). Control (left column) and diet restricted rats (right column). Normal nourished animals had well-limited growth plates with its typical arrangement in cell layers and columns. The diet-restricted animals displayed a thinner and less arranged cell pattern (**B**) Dietary restriction decreased the growth plate area at both end point analysis (p<0.05). (**C**) Growth plate thickness was decreased by undernutrition on both 56 and 70 days (p<0.05). (**D**) On day 70, a significant decrease of the ossified zone was seen in the diet-restricted group (p<0.05). The arrows indicate the growth plate ossified zone. (Hematoxylin and eosin, original magnification x200). Asterisks indicate significant difference (p<0.05).

**Figure 4:**
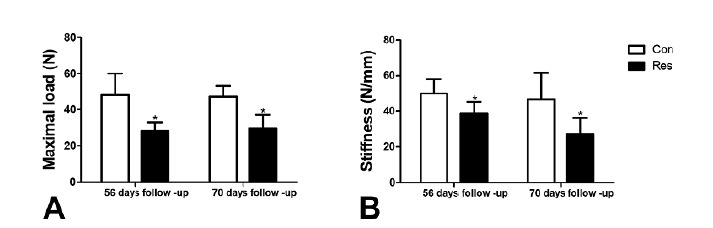
Shearing mechanical testing of the growth plate. The dietary restriction decreased maximal load and stiffness at both end point analysis. Asterisks indicate significant differences (p<0.05).

In agreement with the µCT results, the histomorphometric analysis demonstrated that undernutrition (Res) caused significant detrimental changes in the microanatomy of the growth plate (Fig. 3). On days 56 and 70, undernourished rats exhibited thinner (56 days p=0.02 and 70 days p=0.004) growth plates and lower total area (56 days p=0.04 and 70 days p=0.002). Also, undernutrition weakened the growth plate (Fig. 4) as seen on day 56; there was a reduction of the maximal load (A) by 50% (p= 0.004) and stiffness (B) by 22% (p=0.03). On day 70, these reductions were 40% (p=0.001) for maximal load and 42% for stiffness (p=0.02).

###  Effects of undernutrition on bone tissue 

**Figure 5:**
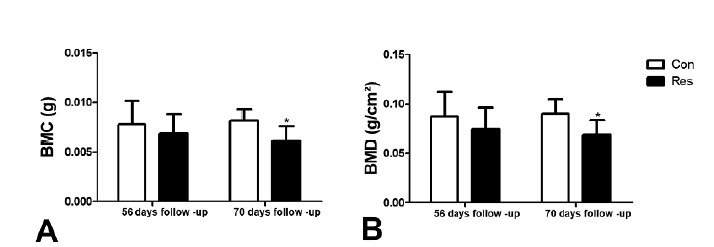
DXA assessments of tibias: (**B**) BMD (g/cm2) and (**A**) BMC (g) of the proximal metaphysis on days 56 and 70. Dietary restriction resulted in decreased BMD and BMC (only at 70 days of follow-up). Asterisks indicate significant differences (p<0.05).

Figure 5 shows a comparison of BMD (B) (56 days p=0.3 and 70 days p=0.02) and BMC (56 days p=0.4 and 70 days p=0.01). (A) at 56 and 70 days. A significant difference was found in the limit time of 70 days, whence Res group rats had less dense bones.

**Figure 6:**
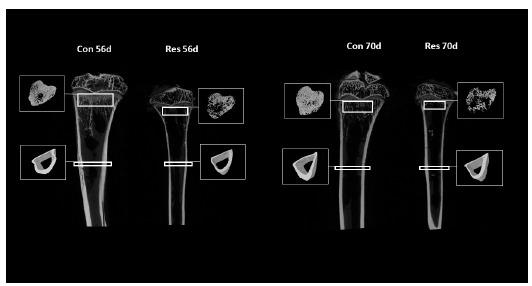
Coronal μCT images of tibias with tridimensional isolated trabecular and cortical bones. Undernutrition resulted in microstructural changes on both trabecular and cortical bones.

The µCT data showed microstructural changes in the trabecular bone associated with undernutrition (Fig. 6). Furthermore, the deleterious changes were found to be time-dependent and were more conspicuous on day 70. Table 2 shows the microstructural parameters. On day 56, the undernourished animals exhibited a decrease in BV by 59% (p=0.02), in BV/TV by 42% (p=0.03), in Tb.N by 38% (p=0.01), and in Conn.D by 76% (p=0.0001), and an increase in Tb.Sp by 39% (p=0.02). On day 70, these changes were more typical; there was a decrease in BV by 76% (p=0.002), in BV/TV by 59% (p=0.008), in Tb.Th by 14% (p=0.01), in Tb.N by 54% (p=0.01), and in Conn.D by 66% (p=0.005), and an increase in BS/BV by 15% (p=0.03).

**Figure 7:**
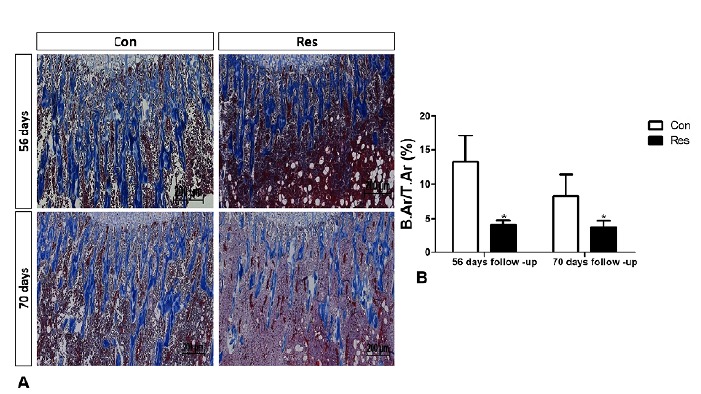
(**A**) Histological coronal sections of the trabecular bone both at 56 and 70 days of malnutrition in control and dietary restricted animals. The Masson staining depicts in blue the trabeculae of bone formed in the region close to the growth plate. (Masson’s trichrome, original magnification x50). (**B**) Histogram showing the amount of trabecular bone in relation to the whole area (B.Ar/T.Ar %) as obtained by morphometric analysis. The diet restriction significantly decreased the area of trabecular bone at both end point analysis. Asterisks indicate significant differences (p<0.05).

**Figure 8:**
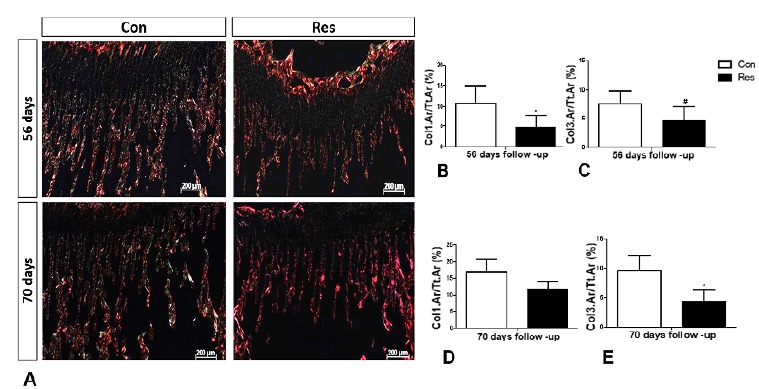
(**A**) Photomicrograph using polarized light. Histological coronal sections of the trabecular bone close to the growth plate evidencing a decrease in collagen deposition in the malnourished rats. The histograms show the percentage of the area of collagen in the region of the trabecular bone. On day 56 they exhibited a decrease both in type 1 (orange red color) (**B**) and in type 3 (yellowish green color) (**C**) collagen levels. No difference was seen in the type 1 collagen level on day 70 (**D**), whereas on day 70, there was a significant decrease in collagen type 3 level (**E**). (Picrosirius red, original magnification x50). Asterisks indicate significant differences (p<0.05) and hashtags indicate p=0.09.

The histomorphometric findings showed that undernutrition resulted in lower trabecular bone mass with lower collagen deposition. In Figure 7, Masson’s trichrome staining indicates lower trabecular bone mass in the diet-restricted group (-69% on day 56 (p=0.005) and, -54% on day 70, (p=0.03). Figure 8 shows representative images obtained for picrosirius red staining, illustrating a decrease in collagen deposition in the undernourished rats. On day 56, a decrease in type 1 collagen levels by 56% (p<0.03) and by 39% in type 3 (p=0.09) was observed. On day 70, the type 1 collagen level decreased by 19% (p=0.2) and by 58% in type 3 (p=0.001).

The cortical bone as studied by µCT also presented microstructural changes associated with undernutrition (Fig. 6), but to a lesser degree than those displayed by the trabecular bone. Similar to that observed in the trabecular bone, the deleterious changes in the cortical bone were also found to be time-dependent, and they were more evident on day 70. Table 2 shows the microstructural parameters. On day 56, the rats that underwent dietary restriction exhibited a decrease in Ct.V by 32% (p<0.0001). On day 70, there was a decrease in Ct.V by 44% (p=0.0001) and in Ct.Th by 14% (p<0.001).

**Figure 9:**
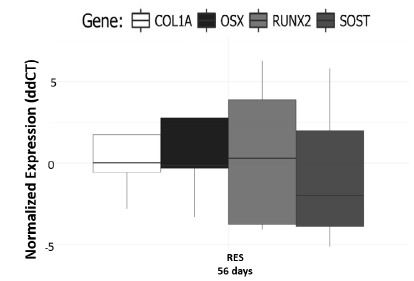
Gene expression analysis. Despite all phenotypic changes in undernutrition, the gene expression of Col1A, Runx2, Osterix and Sos did not differ between the groups (p>0.05).

Despite all the phenotype changes, our PCR data did not show any difference related to gene expression in the diet-restricted rats (Fig. 9). 

## Discussion 

In this study, the effects of undernutrition on the growth plate, trabecular and compact bones of growing rats were investigated by several approaches. Our overall results show that undernutrition causes a harmful impact in weight increase and long bone growth. In our experiment the animals were followed up to 13 weeks of age but were still growing when they were euthanized^20^. Therefore, our results do not permit extrapolation of data to the end of the skeletal maturity, nor if these changes are reversible because we did not introduced a normal diet to the undernourished rats. However, from clinical studies it is known that undernutrition may cause irreversible changes not only in the skeleton, but also in all organs and systems during all life^21^ ^-^ ^24^.

The growth plate, the specific target of this investigation, was greatly affected in the diet restricted animals, both in its structure and function. The basic findings that support this interpretation are a reduction in the thickness of the growth plate in its mechanical weakening, and gross alterations of the microscopic anatomy. Indirect evidences that the function of the growth plate was also disturbed are the poor quality of the lamellar bone, as well as a reduction in length, mass, and diameter of the whole tibia. The unexpected finding was the non-alteration of gene expression. Such findings will be discussed subsequently. 

First, our results indicate that the severe changes in the bone components were time-dependent. The growth plate impairments were related to anatomical changes in its structure (area, height, and tridimensional volume) and strength (maximal shearing load and stiffness). These detrimental changes may have significantly contributed to the bone changes, but we cannot discriminate them from changes that normally occur as a consequence of bone remodeling. Therefore, reduction either in the quantity or function of the cells resulted in less bone formation. The poorer bone quality is expressed by the µCT findings such as decreased trabecular number, connectivity, and density, increased trabecular separation and specific bone surface, and lower collagen deposition (types 1 and 3). Our results agree with previous findings that bone keeps growing even under adverse conditions of undernutrition; however, this results in thinner and weaker cortices as well as less bone mass and length^25^.

Previous studies have also investigated the effects of malnutrition on skeletal tissue^2^ ^,^ ^5^ ^,^ ^9^ ^,^ ^26^ ^,^ ^27^, but not undernutrition as we did. Most of them investigated the depletion of specific nutrients, such as proteins^12^, vitamin D^15^, calcium^14^, and zinc^1^. Considering that most of the cases of clinical undernutrition include reduction in food intake, we chose to use this model in our study. Previously, authors have also studied the effects of the general reduction in food intake either on growth plates^27^ or bones^9^ ^,^ ^26^. However, they did not assess both the skeletal tissues at the same time, and did not use a methodology that assessed the several morphological changes as we did. Pando *et al.* ^2^ combined the study of both tissues and evaluated the effects of 40% dietary restriction on growth plate, trabeculae, and cortical bone. Nevertheless, the growth plate was only assessed for its height by the authors. They found that dietary restriction induced a decrease in growth plate height, and changes in trabecular bone (decrease in volume, number and collagen deposition, associated with an increase in separation and osteoclast number) and cortical bone (thickness and area). But, the abovementioned authors only followed-up the animals for 36 days, where the peak bone mass had not been achieved yet. In the present study, we followed the animals during the main growth period until they achieved the peak bone mass. Pando *et al.* ^2^ analyzed serum bone markers and found a decrease in levels of leptin, insulin-like growth factor 1 and alkaline phosphatase, and all markers for bone formation. Nonetheless, the authors did not assess the molecular mechanisms leading to cartilage and bone changes due to dietary restriction. In our study, we evaluated gene expression to highlight the mechanisms leading to all the detrimental phenotypic changes we observed. We hypothesized that undernutrition downregulates the expression of type 1 collagen (*Col1a1*), Runt Related Transcription factor 2 (*Runx2*), and osterix, since their roles in endochondral bone formation are very well documented in literature^28^ ^-^ ^31^. We did not study the genes of growth cartilage, but those related with the newly formed bone. Despite our histomorphometric data evidencing the lower deposition of collagen and fewer trabeculae in the metaphysis of tibias in diet-restricted rats, we did not find any significant difference related to gene expression. Our unpublished data reveal that *Col1a1* and *Runx2* were downregulated in bone calluses of malnourished rats on day 14 post-fracture. Consequently, studies should then be carried out to investigate the effects of food restriction on gene expression in earlier stages (i.e., 7 or 10 days). 

Our work has some limitations. First, we did not study the effects of undernutrition until the end of the growth, neither whether the pathological skeletal changes persisting along life. Do they persist when a normal diet is introduced? Observations in human beings show that a permanent damage of undernutrition extends irreversibly to adult life, even after refeeding, although a catch up is found after the introduction of a normal diet^5^. Another point is that we did not study the gene expression profile in the growth plate itself. But in our design we did study the gene expression in the metaphyseal bone situated close to the growth cartilage as this newly formed bone reflects the growth plate activity. However, more genes could be studied.

## Conclusions

 50% dietary restriction had detrimental effects on growth plate, trabecular bone, and cortical shaft bone with anatomical and functional repercussions. Although undernutrition did not result in changes in expression of the genes studied, several phenotypic changes were seen in the growth plate. 
